# AAPM MEDICAL PHYSICS PRACTICE GUIDELINE 5.b: Commissioning and QA of treatment planning dose calculations—Megavoltage photon and electron beams

**DOI:** 10.1002/acm2.13641

**Published:** 2022-08-10

**Authors:** Mark W. Geurts, Dustin J. Jacqmin, Lindsay E. Jones, Stephen F. Kry, Dimitris N. Mihailidis, Jared D. Ohrt, Timothy Ritter, Jennifer B. Smilowitz, Nicholai E. Wingreen

**Affiliations:** ^1^ Aspirus Health Wausau WI USA; ^2^ University of Wisconsin Madison WI USA; ^3^ MedPhysLindsay Baltimore MD USA; ^4^ MD Anderson Cancer Center Houston TX USA; ^5^ University of Pennsylvania Philadelphia PA USA; ^6^ VCU Health System Richmond VA USA; ^7^ AAPM Alexandria VA USA

**Keywords:** Dose calculations, MPPG, practice guideline, treatment planning

## Abstract

The American Association of Physicists in Medicine (AAPM) is a nonprofit professional society whose primary purposes are to advance the science, education, and professional practice of medical physics. The AAPM has more than 8000 members and is the principal organization of medical physicists in the United States. The AAPM will periodically define new practice guidelines for medical physics practice to help advance the science of medical physics and to improve the quality of service to patients throughout the United States. Existing medical physics practice guidelines will be reviewed for the purpose of revision or renewal, as appropriate, on their fifth anniversary or sooner. Each medical physics practice guideline represents a policy statement by the AAPM, has undergone a thorough consensus process in which it has been subjected to extensive review, and requires the approval of the Professional Council. The medical physics practice guidelines recognize that the safe and effective use of diagnostic and therapeutic radiology requires specific training, skills, and techniques, as described in each document. Reproduction or modification of the published practice guidelines and technical standards by those entities not providing these services is not authorized. The following terms are used in the AAPM practice guidelines:

- Must and Must Not: Used to indicate that adherence to the recommendation is considered necessary to conform to this practice guideline. **While must is the term to be used in the guidelines, if an entity that adopts the guideline has shall as the preferred term, the AAPM considers that must and shall have the same meaning**.
- Should and Should Not: Used to indicate a prudent practice to which exceptions may occasionally be made in appropriate circumstances.

**Acronyms/abbreviations**
AAPM: American Association of Physicists in Medicine
C/S: Convolution/superposition
CC: Collapsed cone
CT: Computed tomography
DTA: Distance to agreement
GBBS: Grid‐based Boltzmann transport equation solver
IGRT: Image‐guided radiation therapy
IMRT: Intensity‐modulated radiation therapy
linac: Linear accelerator
MC: Monte Carlo
MLC: Multileaf collimator
MPPG: Medical Physics Practice Guidelines
MU: Monitor unit
OAR: Organ at Risk
PB: Pencil beam
PDD: Percent depth dose
QA: Quality assurance
QMP: Qualified medical physicist
SRS: Stereotactic radiosurgery
SSD: Source‐to‐surface distance
TLD: Thermo‐luminescent dosimeter
TPS: Treatment planning system
VMAT: Volumetric‐modulated arc therapy

## 1 INTRODUCTION

The treatment planning system (TPS) is an essential component of external beam radiation therapy. TPSs are used to plan the beam arrangements, energies, field sizes, fluence patterns, and modifiers that provide optimum dose distributions to treat disease and minimize dose to the healthy tissues. The accuracy of the dose calculations is paramount for safe and efficacious treatment delivery. A substantial (but not exclusive) part of commissioning a TPS is ensuring that the radiation beam parameters, and other data affecting the accuracy of the dose calculation, are adequately modeled in the system, and are properly validated. These tasks are the subject of this Medical Physics Practice Guideline (MPPG).

The recommendations in this report are based on the minimum requirements for well‐established commercial systems with available manufacturer's guidance on the commissioning process. The goals and scope of this document are defined below. The Chair of AAPM Medical Physics Practice Guideline Task Group 341 has reviewed the required Conflict of Interest statement on file for each member and determined that disclosure of potential conflicts of interest is an adequate management plan. Disclosures of potential conflicts of interest for each member are found at the close of this document.

### 1.1 Goals

A qualified medical physicist (QMP) is responsible for the commissioning and quality assurance (QA) of TPSs in a clinical radiation therapy department. This document is part of a series of Medical Physics Practice Guidelines (MPPG) commissioned by the American Association of Physicists in Medicine (AAPM) and intended to succinctly state the minimum acceptable standards for various aspects of clinical medical physics.

**FIGURE 1:**
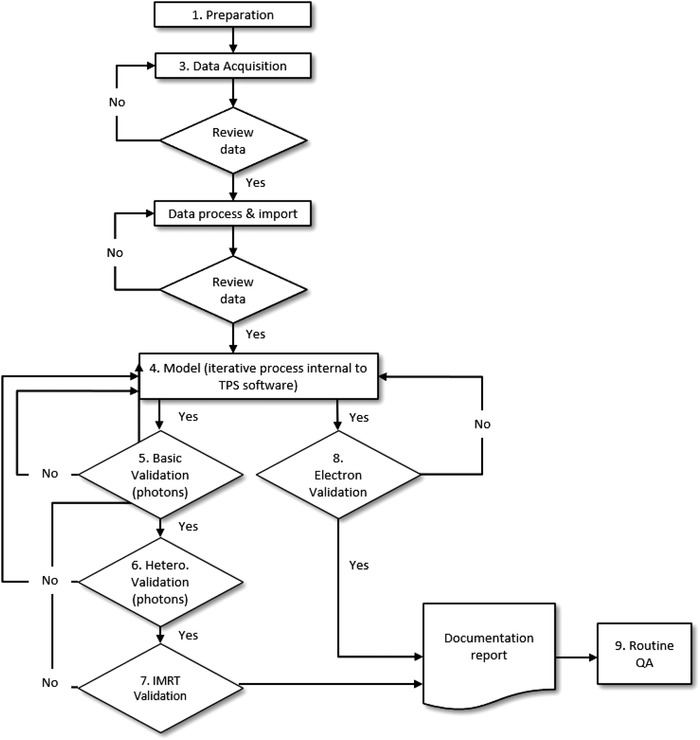
Workflow of TPS dose algorithm commissioning, validation, and routine QA. The numbers refer to sections of this report

Many guidelines, task group reports, and other peer‐reviewed journal articles have been published on the topic of TPS commissioning, evaluation, and QA.^1^, ^2^, ^3^, ^4^, ^5^, ^6^ TPS vendors may provide detailed manuals for their systems. Although the implementation of robust and comprehensive QA programs recommended in other AAPM reports is strongly encouraged, the overall objective of this MPPG is to provide an overview of the minimum requirements for TPS dose algorithm commissioning and QA in a clinical setting. In this report, the term “commissioning” includes beam data acquisition, modeling, and verification. Routine QA and validation tests following a software or hardware update affecting the dose algorithm are subsets of this work and are therefore also covered by this report. Figure 1 depicts activities that are part of commissioning. The specific goals for this report are to:

1. Clearly identify and reference applicable portions of existing AAPM reports and peer‐reviewed articles for established commissioning components.
2. Provide updated guidelines on technologies that have emerged since the publication of previous reports.
3. Provide guidance on validation tests for dose accuracy and constancy (select downloadable datasets/contours and beam parameters are provided for optional use).
4. Provide guidance on tolerance values and evaluation criteria for clinical implementation.
5. Provide a checklist for commissioning processes and associated documentation.

### 1.2 Tolerances values and evaluation criteria

Modeling the commissioning data in the TPS is an iterative process that includes compromises in accuracy over the range of clinical scenarios that could be encountered. Consequently, some aspects of the validation tests may show excellent agreement, whereas others may show poorer agreement. Accurate model verification is affected by both measurement and model limitations. Some components of the dose distribution may be difficult to measure accurately (e.g., detector overresponding to low‐energy photons in the low‐dose tail profiles), whereas, in other circumstances, the TPS may not model the dose well even when appropriate and accurate input data are used.^7^, ^8^ The desired accuracy should be driven by the needs of the clinic. The tolerance values and evaluation criteria in this MPPG represent a compromise between several factors:

1. Avoiding values that are too “tight” and may be unreasonable or unachievable over the investigated range of field sizes, depths, off‐axis positions, test setups, and beam modifiers.
2. Avoiding values that are too “loose” and could therefore result in approval of a suboptimal model.
3. The need for a simple, generic set of evaluation criteria, as opposed to a complex matrix of test scenarios and tolerances for different parts of the model that could potentially lead to confusion.

Each validation section (basic photon, heterogeneity, intensity‐modulated radiation therapy [IMRT]/ volumetric‐modulated arc therapy [VMAT], and electrons) has its own criteria. Although the basic photons are often modeled (and verified) first, it is important to note that if the model just meets the basic photon tolerance values, it will likely provide unacceptable results when IMRT/VMAT evaluation criteria are considered. Parameter values will likely need to be adjusted for IMRT/VMAT modeling but not to the extent that it would change the passing of the basic validation tests; nonetheless, all changes must be validated.

The tolerance values for the basic photon tests are widely accepted for static photon beams under conditions of charged particle equilibrium. The tolerances for the simple heterogeneity and basic electron beam validation tests are also considered widely accepted and therefore are stated as minimum tolerances. However, given that there are no widely accepted minimum tolerance values for many of the IMRT verification tests in this MPPG, those evaluation criteria must not be interpreted as mandatory or regulatory tolerances. Rather, they are values defined as points for further investigation, possible improvement, and resolution. All the tolerances and criteria in this report are based on a combination of published guidelines, the dosimetric audits performed by the Imaging and Radiation Oncology Core—Houston (IROC Houston; formerly the Radiological Physics Center, RPC), and the experience of the authors. Users are encouraged to not only meet these tolerances, but also strive to achieve dosimetric agreement comparable to that reported in the literature for their algorithm(s).

### 1.3 Scope

The scope of this report is limited to the commissioning and QA of the beam modeling and calculation portion of a TPS where:

1. External photon and electron treatment beams are delivered at typical source‐to‐surface distance (SSDs) using a gantry‐mounted radiation source including conventional and small fields used in IMRT, VMAT, and helical tomotherapy delivery.
2. Modern dose algorithms are utilized, including corrections for tissue heterogeneity. For minimum tolerance values and evaluation criteria, this report assumes use of model‐based photon dose algorithms with 3D heterogeneity corrections including convolution/superposition with point kernels, grid‐based Boltzmann transport equation solvers, or Monte Carlo algorithms.^9^, ^10^, ^11^ For electron beams, pencil beam or Monte Carlo dose algorithms are assumed. However, the commissioning process and validation tests should be applied to all external photon and electron beam algorithms in clinical practice at a given facility.
3. The multileaf collimator (MLC) is used as the primary method of shaping the photon beam aperture or modulating the fluence for treatments.

Areas of treatment planning commissioning and QA that fall outside the scope of this report include noncommercial planning systems, those used for stereotactic radiosurgery (SRS), secondary monitor unit validation and other such ancillary software, optimization and leaf sequencing algorithms, methods involving biological modeling (including tumor control and normal tissue complication probability), and all nondosimetric components of the planning system. Nondosimetric components include (but are not limited to) dataset management and presentation, coordinate systems, image generation, image registration, anatomical structures, and functions dependent on anatomy (e.g., dose‐volume histograms, beam's eye view displays).

### 1.4 Intended users and precautions

The intended user of this document is the QMP. Hospital and clinic administration are also encouraged to use this report as a reference for an explanation of process, time, and resource requirements.

This document does not contain specifics on the use of commercially available TPS software. The QMP must be properly trained in the use of the planning software and related systems prior to clinical implementation. In addition, the configured treatment machine type and the planning system should have a history of compatibility and agreement between calculated and delivered dose.

## 2 STAFF QUALIFICATIONS AND RESPONSIBILITIES

The QMP as defined in AAPM Professional Policy 1 must be competent to practice independently in the subfield of Therapeutic Medical Physics. The policy can be accessed online at http://www.aapm.org/org/policies.

A given TPS may include multiple dose calculation algorithms. Prior to beginning commissioning, the QMP must select a dose computation algorithm(s) for commissioning. The QMP must have a clear understanding of the algorithm(s) chosen. The QMP must also review and understand the vendor's guidance documentation on commissioning measurements, to include how the measurements relate to the model parameters and impact the resulting dose distributions.

The QMP should develop a schedule to acquire data and model and verify the dose algorithms. Assuming 12–16 QMP work hours per day (1.5–2.0 FTEs), reasonable time estimates are two to four weeks for a single energy photon beam and 6–8 weeks for two photon energies and five electron energies.^4^, ^5^ This will depend strongly on how much commissioning data need to be collected and the availability and experience of the QMP(s) involved, the adequacy and availability of the equipment used, and the access to the accelerator. Addition of a second algorithm for a given beam will further increase commissioning time and effort. Completing the validation tests in this report has been reported to take approximately 80 hours for one machine and algorithm^12^ with four photon and five electron energies and is consistent with this task group's experience. This process should not be rushed; numerous future treatments will depend on the accuracy of the TPS.

## 3 DATA ACQUISITION AND PROCESSING

This section describes the methods of acquiring the data necessary for modeling a treatment beam. The linac configuration and performance should be tuned and accepted prior to taking any measurements for commissioning, including validations of jaw position and field size. The QMP should understand the details of the required modeling data and should follow the recommendations from the TPS vendor for the required dataset, including the number of and range of field sizes to be measured. The authors of this report strongly discourage reducing the required dataset because of time or convenience. If the TPS is being commissioned in parallel with the commissioning of a new linear accelerator, then a full set of new modeling data is required. If a new TPS and/or new algorithm are being commissioned on an existing linear accelerator, then existing data could be used, provided that they are verified (compared with recent QA measurements to assess any changes in beam characteristics) and meet vendor requirements. However, additional data may also be required. It may be useful to acquire data that will be used for validation (Sections 5, 6.2, 7, 8 of this report) at the same time commissioning data are collected.

### 3.1 Equipment

The QMP should identify the required equipment in advance. The QMP must verify the functionality, correct operation, and calibration status (if applicable) of equipment and understand the limitations and uncertainties of each device regarding the intended measurement. Table 1 summarizes some of the detectors appropriate for use to obtain the data under various conditions. Not all detectors are necessary, provided that an appropriate detector is identified for each task. Table 2 lists the minimum required additional equipment for a typical commissioning effort.

**TABLE 1:** Detectors suitable for TPS commissioning and validation of photon and electron beams

Detectors	Uses	Comments	References
Scanning ion chambers	Beam scanning for photons and electrons	Typical scanning chambers have an air cavity of 4–6 mm diameter	TG‐106 ^4^
Electron diodes and film	Beam scanning for electrons, output factors	Diodes are recommended over ion chambers with electrons to reduce stopping power dependency	TG‐25,^13^ TG‐70 ^14^
Small field detectors	Small field scanning & output factors,^a^ IMRT/VMAT point measurement, MLC intraleaf measurement, & penumbra	Carefully select the detector type and size to fit the application. When scanning for penumbra, diodes are recommended.	TG‐106,^4^ TG‐120,^15^ TG‐155,^16^ IAEA TRS‐483^17^
Large ion chamber	Aggregate MLC transmission factors	Interleaf transmission	LoSasso *et al*.^18^
Film and/or array detector	2D dose distributions, including dynamic/virtual wedge and planar fluence maps, intraleaf measurements^b^	Absolute dosimetry preferred, relative dosimetry adequate. Desirable if the device can be mounted on the gantry and/or in a phantom at different geometries	TG‐106,^4^ TG‐120,^15^ IAEA TRS‐430 ^3^

^a^ If a diode detector is used for small field measurements, a “daisy chain” approach is recommended to minimize the energy‐dependence effects; the diode is first cross‐compared with an ion chamber for a larger field and then is used to measure the smaller fields.

^b^ Using film for intraleaf transmission is usually less precise than interleaf transmission.

**TABLE 2:** Equipment required for TPS commissioning of photon and electron beams

Equipment	Uses	Comments	References
3D water phantom	Beam scanning	Must have sufficient scanning range and lateral/depth scatter	TG‐106,^4^ TG‐70 ^14^
Electrometers and cables	Beam scanning, output calibration, relative and absolute dosimetry	ADCL calibration, low noise and leakage with wide dynamic range and linear response	TG‐51,^19^ TG‐106 ^4^
Buildup cap or mini phantom	In‐air output factor measurement	Measurements required for some planning systems, some second check systems	TG‐74,^20^ TG‐312 report to be published
Water‐equivalent phantom material in slab form	Buildup and backscatter for measurements	>20 cm of total thickness in varying increments, width and length >30 cm, cavity for detector(s)	TG‐106,^4^ TG‐120,^15^ IAEA TRS‐430 ^3^
CT density phantom	CT number to electron or mass density calibration	Should include tissue‐equivalent materials spanning the clinical range of low‐density lung to high‐density bone.	TG‐66 ^21^
Array detector	Nonphysical wedge measurement and other 2D dose distributions	The array detector should be calibrated for each energy it is used for.	TG‐120^15^
Heterogeneity phantom with lung‐equivalent material	End‐to‐end testing	Include cavities for detectors, useful for annual QA reference test	TG‐65,^22^ IAEA TRS‐430 ^3^
Anthropomorphic phantom	Anatomic model testing, end‐to‐end testing, use testing	Include cavities for detectors	IAEA TRS‐430 ^3^
Software for data processing	Processing, comparing, and analyzing profiles, depth‐dose curves, and other beam data	May be included with the 3D water tank scanning software	TG‐106 ^4^
IMRT/VMAT or arc therapy phantom	VMAT or arc therapy	Options include a solid phantom holding a planar array, 3D detector arrays, film inside a phantom, other	TG‐120 ^15^

The QMP must be aware of setup variables and measurement uncertainties associated with beam data collection. Choices, such as scanning speed, detector size, noise, data processing, detector orientation, and a myriad of other factors, can significantly alter the measured results. The QMP must be aware of and account for the effective point of measurement of ionization chambers used for data collection. Task Group (TG) 106^4^ provides an excellent summary of these topics and should be referenced for additional details. The achievable level of accuracy should also be considered prior to beginning the commissioning process, as this will affect equipment choices and measurements.

### 3.2 Data acquisition for CT calibration

The dose calculation model will typically be commissioned based on dose measurements made in water and air. Dose calculation in heterogeneous media is dependent on the correct mapping of voxel intensities in a CT scan to some physical descriptor that can be used in the algorithm; typically physical or electron density, or less commonly chemical composition,^23^ in the form of a “CT‐density” table.^21^

The QMP must consider the range of clinically relevant material densities and CT acquisition parameters (kVp) as important components of the dose algorithm commissioning process. Materials used for CT number mapping must range from air (≈ 0.001 g/cm^3^) to high‐density material (≈ 2 g/cm^3^), including densities to mimic lung (≈ 0.3 g/cm^3^) and dense bone (≈ 1.4–1.9 g/cm^3^). High‐density calibration points (such as gold or titanium) may also be required. The user must be aware of the bit range used for characterizing CT number and avoid mapping materials that exceed the usable ceiling, which can happen when high‐density metals are mapped using only a 12‐bit depth. Image data should be evaluated over a large volume of each density plug to determine an average CT number for each density. A separate CT density curve should be developed and validated for the image guidance system if those CT datasets will be used for dose calculation. It is recommended that CT scanner‐specific calibration curves be obtained.

### 3.3 Data acquisition for IMRT/VMAT delivery

The acquisition of data for conventional beam modeling is well documented.^4^ This report expands those recommendations to address the additional measurement considerations when modeling small beam apertures and MLC parameters characteristic of IMRT/VMAT delivery. The challenges of small field dosimetry have been well documented and require extremely careful measurement setup and use of an appropriate detector.^24^, ^25^, ^26^ Low et al.^15^ provide an overview of IMRT dosimetry instruments and methods.

Dosimetry for small fields is often extrapolated by the TPS. The MLCs, which define the small fields, also display considerable design variation between manufacturers.^27^ Therefore, measurements to verify both small fields and MLC characteristics are crucial to IMRT/VMAT dose calculation accuracy.

1. Even if not specified by the TPS vendor, the QMP should measure percent depth dose (PDD) with a small‐volume detector down to a field size of 2 × 2 cm^2^ or smaller for comparison with dose calculation.
2. Vendor recommendations for measuring MLC intraleaf and interleaf transmission and leaf gap should be followed using a large detector if an average intra‐ and interleaf value is specified. For separate measurements, a small chamber should be used under the leaf and film should be used for interleaf leakage measurements.^18^
3. Leaf‐end penumbra should be obtained with a small detector (such as a diode or microchamber) to avoid volume‐averaging effects.
4. Leaf timing for binary MLC systems should be verified using film or exit detector measurements.^28^
5. Small‐field output factors (down to 2 × 2 cm^2^ or smaller) should be measured for beam modeling and/or verification.^25^, ^29^

Regarding small‐field measurements, AAPM TG‐155 describes appropriate detector selection and methods.^16^ The threshold for considering these special conditions depends upon the dimensions of the detector being used for small‐field measurement and the lateral charged particle equilibrium range in the medium, a quantity referred to as the $r_{LCPE}$ in the report.

Table 3 shows approximate values of $r_{LCPE}$ for typical clinical photon energies using equation (1) from Palmans et al.^30^ A detector is appropriate for central axis measurements if the half‐width of a radiation beam is greater than the sum of the $r_{LCPE}$ for the beam plus half of the largest external dimension of the detector. This can be expressed using the following equation,^17^ where FWHM is the full width half maximum of the field and *d* is the largest external dimension (e.g., the larger of the length or diameter for a cylindrical chamber) for the detector being used:

$$FWHM\geq 2r_{LCPE}+d.$$

**TABLE 3:** Approximate values of r_LCPE_ for typical clinical photon energies. The FWHM values represent the smallest field size that can be confidently measured for two representative detector sizes without special small field dosimetry considerations or verifications

%dd(10,10)x	Representative Photon Energy (MV)^a^	$r_{LCPE}$ (cm)	Minimum FWHM for representative 7 mm largest external dimension (cm)	Minimum FWHM for representative 3 mm largest external dimension (cm)
66	6	1.0	2.7	2.3
73	10	1.6	3.9	3.5
77	15	1.9	4.5	4.1
80	18	2.1	4.9	4.5
63	6FFF	0.8	2.3	1.9
71	10FFF	1.4	3.5	3.1

^a^ FF refers to a flattening filter free beam.

In addition to measurement considerations, there are other challenges in modeling small‐field output factors. Problems have been observed in terms of TPS calculation accuracy, particularly in the case where small MLC fields lie within larger jaw‐defined fields. Cases have been observed where small fields have been found to have errors in output in excess of 10% or even 20%.^31^, ^32^

### 3.4 Review of data

All data used in the modeling process must be reviewed both before and after entry into the planning system. There are three recommended components to this review.

1. Acquired data must be reviewed for potential setup and measurement errors prior to importing data into the TPS. Inverse square effects, beam divergence, expected beam energy changes with field size, and other well‐known characteristics should be validated (this is often easily performed by review of graphical display of the results). Crossbeam profiles at varying depths and field sizes can be superimposed on the same plot to identify trends. Depth‐dose plots can be analyzed in a similar fashion.
2. The data should be compared, if possible, to a reference dataset from the same type of, or a nearly identical, machine to identify systematic anomalies in either setup or machine properties. Points representing the middle, as well as extremes, of the data should be validated in this manner. Tolerance levels for this step cannot be provided because each machine is unique; however, the mean and range of values for a large number of accelerator types are available in the literature^5^, ^33^, ^34^, ^35^ with evaluations of parameters that present the largest challenges for modeling.^34^, ^36^
3. After the data are entered into the planning system, they must be reevaluated for potential processing errors (e.g., problems during import, smoothing, mirroring). A combination of graphical review and spot‐checking can be used.

## 4 MODEL WITHIN TPS SOFTWARE

Once the measured data and machine parameters are entered into the modeling module of the TPS, the actual beam modeling should be completed according to vendor instructions. For some TPSs, the linac vendor provides a predefined model or performs the modeling using customer supplied data; however, the validation process is still necessary, and the vendor should provide reference data for comparison with QMP measurements.

Modeling is an iterative process, with parameters adjusted to optimally agree with the data used for comparison. The amount of adjustment available to the user varies between TPS vendors. Regardless of how much latitude exists in adjusting parameters, the QMP must understand how the measurements relate to the model parameters, and how each one (and its magnitude) will affect the resulting dose distribution. The QMP must understand the trade‐offs; the model is just that, a “model,” and will therefore not fit the measured data under all measurement conditions with the same accuracy. The QMP should evaluate the goodness of the fit based on qualitative assessment of the dose distribution (PDD and profiles) and use quantitative metrics within the modeling software.

After assessing the quality of the modeling within the TPS beam commissioning (or physics) application, this report recommends additional tests to validate the dose calculations (Sections 5, 6.2, 7, 8). The results from each test should be used to adjust the model (or tune the machine in the case of matched or “twinned” systems), as needed. Sections 5, 6.2, 7 should be carried out in order, meaning that the basic validation testing in homogeneous media should be completed prior to testing in heterogeneous media.

The IMRT/VMAT dose is usually the last photon validation process. It is important to note that there are special considerations for modeling the MLC that strongly affect the algorithm's ability to correctly compute dose from an aggregate of small fields as used in IMRT/VMAT. Leaf transmission and/or dosimetric leaf gap offset can often be used to improve agreement between measured and calculated dose. Therefore, if changes are made to basic photon parameters in the iterative IMRT/VMAT modeling process, the basic photon validation must be reconfirmed. MLC parameters should be compared to the published results obtained with the same MLC and energy.^15^

V10endors of certain systems such as TomoTherapy®, ViewRay®, Radixact®, Unity®, and Halcyon® may provide the user with a preconfigured TPS and provide no means for the physicist to alter the model parameters. In these situations, thorough validation using this report is still important. The QMP should research and review published experiences and recommendations from other clinics^37^ and compare results to published experiences.^38^, ^39^, ^40^

## 5 PHOTON BEAMS: BASIC DOSE ALGORITHM VALIDATION

The basic photon beam dose validation tests described in this section must be completed for each configured beam. A “configured beam” is typically distinguished as a unique energy and/or accelerator head model configuration. For example, 6 MV, 6 MV SRS, and 6 MV flattening filter‐free (FFF) beams are all considered unique beams. Each physical wedge is a unique beam because of its independent energy fluence spectrum and therefore must be separately validated. Nonphysical wedges can be considered an extension of the corresponding open field, and only one additional validation test is presented in this report (described below). The typical setup for the measurement of basic algorithm validation tests will be a static gantry angle pointing directly down with collimator rotated as needed to acquire the appropriate data. If there are multiple algorithms commissioned for a configured beam, each algorithm must be independently validated.

Much of the validation data can be acquired using a scanning water phantom; however, this task group considers an array detector with solid water appropriate for the subset of tests that can be evaluated with a planar detector (e.g., profiles). Validation plans should be created by the clinical users of the system and should exploit typical clinical processes. Although it is good practice to use field configurations for validation that were not used for modeling for many of the tests, it is efficient to collect the validation data at the same time as the modeling data are acquired.

### 5.1 Validation tests

The dose algorithm validation consists of two parts: checks that only require dose computation and checks that compare computed doses to measurements.

Table 4 summarizes three TPS modeling checks for which no additional measurements are required. The objective of each check, example methods for accomplishing it, and dose comparison tolerances are provided. For test 5.1, a beam equivalent to the beam calibration geometry (e.g., 10 x 10 cm^2^, 100 cm SSD or SAD) should be planned in the TPS to ensure that the dose per MU matches the measured value under TG‐51^19^ measurement conditions at the calibration depth (e.g., 10 cm depth). This should be tested for each beam, and a similar test is performed for electron beams (refer to Section 8). Test 5.2 is intended to confirm that the dose calculated to the water phantom in the modeling and clinical planning modules are identical within statistical uncertainty (for Monte Carlo algorithms) or material definition (when different materials exist between modules). This comparison should be performed using a large field for which commissioning data was acquired. Doses at several points should be confirmed. This test should be performed once per algorithm. Test 5.3 confirms that dose calculated to a water phantom in the clinical planning module matches a subset of input commissioning measurements. Parameters such as PDD, output factors, and off‐axis factors should be compared, for example, on a point‐by‐point basis. Choose depths, off‐axis positions, and field sizes other than those specified as the absolute dose reference (e.g., if the TPS output is specified at 10 cm depth in a 10 × 10 cm^2^ field, calculate and compare the dose off axis at other depths and field sizes). While such a point‐by‐point analysis of basic measured versus calculated values may feel redundant, it is an essential part of validating the quality of the beam model; moreover, such a simple evaluation has, in fact, revealed errors at a majority of facilities visited by IROC.^31^, ^34^, ^36^

**TABLE 4:** TPS modelling checks and tolerances

#	Objective	Examples	Tolerances
5.1	Verify that TPS reproduces the absolute dose under reference calibration conditions	Calculate the absolute dose per MU to the reference point using the same SSD and field size as calibration protocol	0.5%
5.2	Compare calculated dose in clinical planning module to calculated dose in physics module^a^	Compare dose profiles computed in the modeling tool to profiles computed in the clinical planning module for a large (> 30 × 30 cm^2^) field	Within statistical, grid size, and material definition uncertainty
5.3	Perform spot checks comparing calculated dose in clinical planning module to data collected during commissioning	Calculate and compare PDD, output factors, and/or off axis factors for nonreference depths, off axis positions, and field sizes	2%^b^

^a^ Applicable if the treatment planning system has a physics module for beam modeling.

^b^ When comparing profiles such as PDD, this tolerance is the local difference.

Table 5 describes tests that validate key aspects of the TPS and/or beam model not typically considered during modeling. Test 5.4 evaluates the ability of the model to accurately calculate dose in the smallest non‐SRS and non‐IMRT fields that are expected to be encountered during clinical use. On the other extreme, Test 5.5 should challenge the MLC model with large fields where substantial areas of the field may be exposed to leaf transmission and leakage. This field should also evaluate both how the TPS models leave that overtravel fully across the field and how the output factor is affected by substantial MLC blocking. Test 5.6 assesses the model at the edges of the field, especially if the primary collimator is exposed at the largest field sizes. A diagonal profile or rotated collimator may be required to evaluate these conditions. Other potential concerns, such as kernel tilting (or lack thereof), will also become apparent in these conditions and should be evaluated. Test 5.7 validates the accuracy of the TPS at an SSD different from that used for model data acquisition. Evaluating depth dose curves and/or field edges will confirm that the TPS accurately models the distance from the source across the range of expected clinical use. Test 5.8 evaluates the accuracy of the TPS to calculate dose in fields that are oblique to the surface. The authors recommend attempting to achieve at least a 20° incidence for this test. Shifting laterally or increasing the SSD may be needed when performing the corresponding water tank measurements. Alternatively, solid water may allow for greater obliquity. Finally, Test 5.9 validates nonphysical wedges and may be easiest to measure with an array detector.

**TABLE 5:** Basic photon beam validation checks. See Table 6 for tolerances

#	Dose comparison objective	Examples^a^
5.4	Small static (non‐SRS) MLC‐shaped fields	IAEA TRS 430^3^ Photon Test 1
5.5	MLC transmission, leaf overtravel, and output factor effects	Large field with extensive blocking such as mantle, IAEA TRS 430 Photon Test 3
5.6	Off‐axis modeling including primary collimator	Off‐axis field, IAEA TRS 430 Photon Test 2, measure with diagonal profiles or collimator rotation
5.7	Field divergence and depth dose changes with SSD	Asymmetric field at minimum SSD, IAEA TRS 430 Photon Test 6
5.8	Oblique surface incidence	10 × 10 cm^2^ field at 20° incidence, IAEA TRS 430 Photon Test 10
5.9	Nonphysical wedge fluence modifiers^b^	Large (>15 cm) field for each nonphysical wedge angle

^a^ Multiple objectives can be consolidated into one measurement field, such as 5.5 and 5.6.

^b^ 5.4–5.8 are intended for each open and (hard) wedged field. Nonphysical wedges are considered an extension of the corresponding open field in terms of spectra and only require the addition of 5.9.

Tests 5.4–5.8 should be done for each unique beam. Several example fields are described in the IAEA TRS Report 430.^3^ Some objectives may be tested using the same measurement field, such as Tests 5.5 and 5.6.

For all tests, measurements in the high‐dose region, penumbra, and low‐dose tail regions should be compared to calculated values at various depths (including slightly beyond *d*_max_, midrange/10–15 cm, and deep/25–30 cm) and off‐axis positions. Table 6 summarizes the evaluation methods and tolerances for basic photon tests in Table 5. For an inverse planning only TPS (e.g., Precision for tomotherapy and Radixact delivery systems), the basic photon tests can be performed by creating simple targets and optimizing a plan for each case (e.g., small, large, on/off‐axis, variable SSD) or by performing calculations and/or measurements on static fields provided by the vendor.

**TABLE 6:** Basic TPS photon beam evaluation methods and tolerances

Region	Evaluation methods	Tolerances^a^
High dose	Relative dose with one parameter change from reference conditions	2%
	Relative dose with multiple parameter changes^b^	5%
Penumbra	Distance to agreement	2 mm^c^
Low‐dose tail	Up to 5 cm from field edge	3% of max field dose

^a^ Tolerances are relative to local dose unless otherwise noted, and are consistent with those used by IROC Houston.

^b^ For example, off‐axis with physical wedge.

^c^ 2 mm aligns with evaluation tolerances used in TG‐53 ^1^ and TG‐218.^41.^

As discussed in the introduction, TPS modeling is an iterative process that includes compromises in accuracy over the range of clinical scenarios that could be encountered. In the spirit of minimum practice guidelines, these basic photon tolerance values, especially in the situation with multiple parameter changes (e.g., an off‐axis measurement in the presence of a wedge), are the “worst case scenarios.” Some aspects of the tests in Table 5 may show excellent agreement, whereas others may show poorer agreement. This report recommends that the results of the validation tests should meet criteria consistent with those of IROC Houston. These tolerances are summarized in the recommendations below and in Table 6.

### 5.2 Recommendations

1. The calibration reference condition dose per MU should match within 0.5% (Test 5.1).
2. The relative dose distributions calculated by the TPS should match measured values in the high‐dose regions at different depths and off‐axis positions to within 2% for fields with one parameter changed from the reference conditions.
3. For fields with multiple parameter changes (e.g., an off‐axis measurement in the presence of a wedge), disagreement up to 5% is allowed. It is further noted that most validation experiments should display significantly better agreement than 5%, and if a large number of the results are near this tolerance, additional model improvement should be investigated.
4. The penumbra should match with a 2 mm distance to agreement.^1^, ^41^
5. The low‐dose profile tails, up to 5 cm from field edge, computed by the TPS should agree with measurement to within 3% of the in‐field dose.
6. Limitations uncovered during validation testing should be communicated to the clinical treatment team for consideration. For example, if small fields were found to have deficiencies, those field sizes could either be restricted during planning or a decision could be made to perform special measurements.

Users should always strive for the best possible agreement between modeled and measured results. The QMP must understand the limitations of the dose calculation algorithm in measurement conditions such as the buildup region, oblique incidence, and penumbra. Although it may be deemed clinically acceptable for the TPS to disagree with the delivered dose by more than the above criteria, these cases must be understood, clinically justified, and documented. It is important to reiterate that, if the model barely passes the basic photon recommendations on a machine that will also be used for IMRT/VMAT, the dosimetric agreement for IMRT/VMAT plans will likely be poor. It is also recognized that additional modeling for the IMRT/VMAT may affect the parameter results of the basic photon beam modeling, specifically the penumbra and tails. Once IMRT modeling is completed, the basic beam modeling will therefore need to be rechecked. Consequently, the physicist may want to conduct IMRT/VMAT modeling before basic photon modeling is finalized.

## 6 PHOTON BEAMS: HETEROGENEITY CORRECTION VALIDATION

The commissioning of heterogeneity corrections requires the accurate commissioning of the beam itself and accurate characterization of the patient data. For dose calculation in heterogeneous media (e.g., the thorax), modern and advanced algorithms such as convolution/superposition (C/S), collapsed cone (CC), grid‐based Boltzmann transport equation solver (GBBS), or Monte Carlo (MC) are required, and pencil beam (PB) and correction‐based algorithms are unacceptable. Many studies detail the accuracy of these algorithms.^22^, ^42^, ^43^, ^44^, ^45^, ^46^ The QMP must understand not only the implementation of their heterogeneity corrections, but also their limitations, particularly in the context of known dose discrepancies, which should be distinguished from incorrect implementation/commissioning of the TPS. Care should be taken when evaluating calculated dose (1) within low‐density tissue, (2) near the interface of heterogeneous tissues, and (3) beyond low‐/high‐density tissue. A detailed overview of many types of heterogeneity corrections and tests can be found in the AAPM Report 85^47^ and IAEA TRS Report 430.^3^

Compounding transport issues through heterogeneities, different algorithms transport and calculate dose to different media. Some algorithms transport and calculate dose to the material within the voxel, whereas other algorithms essentially treat all materials as water of varying density. It is also possible to convert between the two, in which the dose to medium is converted to “dose to water,” usually through application of stopping power ratios.^48^ However, this stopping power‐based conversion has actually been found to decrease dosimetric agreement with conventional TPS doses in most cases,^49^, ^50^ leading to “dose to medium” being recommended.^49^ The QMP must be aware of which dose is being reported; guidance on this topic is available from TG‐329.^51^

### 6.1 Validation tests

The recommended minimum validation of the heterogeneity calculations includes confirmation of the lookup table, bulk density, or material assignment CT‐density conversion and basic validation of TPS calculations beyond lung heterogeneities. These validations should be performed for all CT scanners used to generate datasets on which dose is computed. This includes on‐board imagers if adaptive dose computations are part of the clinical workflow.

Table 7 summarizes the validation testing for TPS dose calculations in heterogeneous media. Test 6.1 is a simple verification that the TPS‐reported densities match the actual densities of the phantom.^3^ Test 6.2 verifies dose beyond low‐density (lung) material for each beam energy. Any heterogeneous phantom available can be used for these measurements. A reasonable slab phantom setup is found in Carrasco et al.^43^ It consists of a 5 cm slab of water‐equivalent plastic stacked upon a 13 cm slab of lung‐equivalent material, upon a 10 cm slab of water‐equivalent plastic. For lung‐equivalent material, any type of low‐density and low‐Z material, such as a low‐density wood (approximately 0.3 g/cm^3^), can be used, if the thickness is sufficient to result in a dose correction greater than 10% compared to a homogeneous phantom. This test can be easily performed with a static forward planned beam setup; however, for planning and/or delivery systems for which this is not available, a simple inverse planned test can be generated. For example, an inverse plan can be optimized to deliver a uniform dose to a simple 5 cm^3^ target.

**TABLE 7:** Heterogeneous TPS photon beam validation checks

#	Objective	Examples	Tolerances^a^	References
6.1	Validate planning system reported electron (or mass) densities against known values	CT‐density calibration for air, lung, water, dense bone, and possibly additional tissue types	–	TG‐65,^47^ IAEA TRS‐430 ^3^
6.2	Heterogeneity correction distal to lung tissue	5 × 5 cm^2^, measure and calculate dose ratio above and below heterogeneity, outside of the buildup region	3%	IAEA TRS‐430,^3^ Carrasco et al.^43^

^a^ Tolerances are relative to local dose unless otherwise noted.

Regardless of the plan type used for Test 6.2, the ratio of the dose values above and below the heterogeneous medium along the central axis must be measured and compared with the TPS calculated under the following conditions:

1. Measurements should be made outside of the buildup/builddown regions.^22^ This simple test allows for the direct study of the calculation accuracy through the heterogeneity.
2. A small field size, such as 5 × 5 cm^2^, is recommended because discrepancies due to low‐density material tend to be exacerbated at smaller field sizes.^47^

Further tests deemed appropriate by the QMP to challenge the accuracy of the algorithm being employed should be used to bring a better understanding of the limitations of dose calculation in the vicinity of other heterogeneities. This may include measurements in the presence of higher density materials to which the QMP has access, such as slabs of bone equivalent density or metal implants.^52^

### 6.2 Recommendations

1. To produce acceptable dosimetric accuracy in highly heterogeneous media (particularly in lung), an algorithm comparable to C/S, CC, MC, or GBBS‐based dose calculation algorithm must be used.
2. The QMP should understand the implementation and limitations of the heterogeneity corrections used in the chosen algorithm.
3. The CT to density lookup table and/or bulk density or material assignment process, as discussed in Section 3.2, should be used to accurately construct a CT‐density table within the TPS and should be verified (Test 6.1). This validation should include on‐treatment imaging modalities (MVCT, CBCT, CT on rails, etc.) if they are used for dose calculation.
4. The impact of low‐density heterogeneities on central axis dose should be quantitatively verified with a recommended 3% dose agreement beyond lung‐equivalent material (Test 6.2).

## 7 PHOTON BEAMS: IMRT/VMAT DOSE VALIDATION

This section describes the comparison of the individual beam and/or composite measurements of IMRT/VMAT/helical delivery plans with TPS calculations. Despite widespread IMRT utilization, accurate dosimetric commissioning of an IMRT system remains a challenge. Current IMRT phantom pass rates from IROC Houston^53^ show that only 90% of institutions passed the end‐to‐end anthropomorphic head and neck phantom test with a lenient dose‐ratio and distance‐to‐agreement (DTA) criteria of 7% and 4 mm, respectively. Only 70% of the irradiations passed narrowed criteria of 5% and 3 mm.^53^ A substantial fraction of the failures was traced to the fundamentals of the TPS commissioning. As such, the approach and acceptance criteria used for dosimetric commissioning of IMRT/VMAT are of paramount importance.

Equally important to correct modeling is correct validation. Numerous studies have found that current techniques to evaluate IMRT plan accuracy have very poor sensitivity. That is, many of the most common devices do not flag plans that have major errors.^54^, ^55^, ^56^, ^57^, ^58^, ^59^, ^60^, ^61^ For example, of those plans that failed IROC phantom criteria (and showed large systematic dose differences), the institution's assessment of the plan showed extremely high gamma pass rates with their own system.^58^, ^62^ Therefore, it is essential that validation measurements be conducted with extreme caution. The most common error seen in IROC phantoms is systematic dose deviations,^53^ and these errors are substantially associated with errors in beam modeling.^63^ Therefore, it is critical to not allow systematic differences to be missed during validation. Absolute dosimetry should be used to confirm the dose distribution and can be conducted with simple ion chamber measurements in the high and moderate dose regions.

IMRT/VMAT validation is often performed iteratively while fine tuning the model. Once completed this process should result in two outcomes: first, the QMP should have established tolerance criteria and baseline results for future patient‐specific QA measurements, and second, the QMP should have identified the accuracy limitations of the model and dose algorithm for each clinical application. For the latter outcome, gamma criteria or other dose comparison methods should use as stringent criteria as needed to identify those limitations. Devices used for validation should ideally be independent from those used to tune the model.

AAPM TG‐218^41^ offers a comprehensive overview and guidance for performing IMRT QA and should be considered when designing these validation tests and tolerances. For an established IMRT QA program, they have recommended gamma criteria of 3%/2 mm/10% (global/DTA/threshold) with tolerance and action limits for patient‐specific pretreatment QA of 95% and 90%, respectively. These values are tighter than the more ubiquitous 3%/3 mm in use by most clinics at the time of the publication of but not as tight at the 2%/2 mm suggested by MPPG 5.a for model evaluation.

### 7.1 Validation tests

In Table 8, there are five types of validation tests recommended for IMRT/VMAT delivery modalities. Once the initial tests plans are developed with the most frequent energy (often 6 MV), the plans can be recalculated for the remaining energies, if applicable. If multiple delivery techniques are available for the same accelerator (e.g., segmental IMRT, dynamic IMRT, VMAT, tomotherapy), each one must be validated separately. As described in Section 5, the FFF version of the same nominal energy beam is a unique, configured beam that requires separate validation.

**TABLE 8:** VMAT/IMRT checks. See Table 9 for tolerances

#	Objective	Examples	Detectors	References
7.1	Verify small field PDD	< 2 × 2 cm^2^ MLC shaped field, with PDD acquired at a clinically relevant SSD	Diode or plastic scintillator	TG‐155^16^
7.2	Verify output for small MLC‐defined fields	Use small square and rectangular MLC‐defined segments, measuring output at a clinically relevant depth for each^a^	Diode, plastic scintillator, mini chamber or microion chamber	TG‐155,^16^ Cadman et al.^64^
7.3	TG‐119 tests	Plan, measure, and compare planning and QA results to the TG‐119 report for both the Head and Neck and C‐shape cases	Ion chamber, film, and/or array	TG‐119 ^65^
7.4	Clinical tests	Choose at least two relevant clinical cases; plan, measure, and perform an in‐depth analysis of the results	Ion chamber, film, and/or array	TG‐218 ^41^
7.5	External review	Simulate, plan, and treat an anthropomorphic phantom with embedded dosimeters.	Various options exist^b^	Kry et al.^66^

^a^ A bar pattern scanned with a diode can be used to obtain additional absolute dose profile comparison in the direction perpendicular to MLC movement ^64.^

^b^ If IROC Houston service is used, they typically employ TLDs and radiochromic film. Certain commercial phantoms can accommodate ion chambers for point dose measurements.

As a predosimetry test of the system, TPS parameters should be compared, where possible, to available data. For example, the community consensus for many TPS parameters on multiple planning system platforms was recently compiled by IROC.^67^ Parameters such as MLC offset and MLC transmission should be compared to the community distribution. It is important to note that a value that deviates substantially from the community median does not necessarily represent an error, but extreme values should be double‐checked extensively and reviewed with colleagues or the manufacturer.

Test 7.1 is a verification of small‐field PDD. As mentioned in the data acquisition section, the TPS may not require small‐field depth doses for beam modeling. The verification is important because extrapolated data will effectively be used for planning and computing dose in modulated fields. This can help in understanding the limits of the TPS. If the TPS is provided with a predefined beam model, then the QMP should request reference data for these measurements from the vendor.

Test 7.2 is verification of small MLC defined field output not explicitly used in beam modeling. This test differs from the small‐field MLC basic validation (Test 5.4), which represents a field that could be clinically used on its own. As the gap between opposed leaves can be 1 cm or less in IMRT/VMAT, it is imperative to measure the output of small MLC shaped fields, including IMRT‐type fields where the jaws are substantially more open than the MLC “opening.” The QMP should measure output factors down to a field size of 2 × 2 cm^2^ (and preferably smaller) for a clinically relevant depth, and then compare the measured results to the TPS calculations.^16^, ^29^ The jaws should be positioned to reflect their state during the IMRT/VMAT field delivery. Shapes that are located away from the central axis are common in VMAT and IMRT and should be investigated. Tests 7.1 and 7.2 are intended to be performed in static (nonmodulated) conditions, even though the clinical implementation of such small fields would be used in modulated plans. Even for systems for which nonmodulated fields are not easily generated or used clinically (such as helical tomotherapy or MR‐IGRT only systems), the QMP is encouraged to generate simple IMRT beam/target arrangements or work with vendor to generate nonmodulated fields for these tests.

The remaining test plan strategy follows the progression of modulated plans from simple to more complex clinical implementation. Test 7.3 recommends two plans from the TG‐119 test suite^65^ as a starting point: the mock Head and Neck and C‐shape tests. Test 7.4 recommends using at least two image sets for optimization and delivery verification that are representative of the intended clinical cases to be treated. Each modality (e.g., IMRT, VMAT, tomotherapy) and energy must be separately validated. Users can use their own cases or download sample datasets and objectives from http://www.aapm.org/pubs/MPPG/TPS/. Test plans should use the same dose grid resolution (and angular control point resolution for VMAT) that will be used clinically. Tests 7.3 and 7.4 should also be used to test the patient‐specific QA process. The plans should be delivered to a phantom with appropriate dosimeters that will enable the user to compare planned and delivered dose distributions.^68^

Test 7.5 is a complete end‐to‐end test that involves scanning an anthropomorphic phantom, treatment planning, delivery, and sending dosimeters out for external review. Two such end‐to‐end external validations are recommended. A head and neck plan, such as the IROC Houston credentialing test,^53^ is encouraged as one end‐to‐end test. To test IMRT/VMAT delivery in the thoracic region, a second end‐to‐end test with a heterogeneous thoracic/lung phantom should be performed.^66^ Even with modern model‐based dose calculation algorithms, systematic differences between calculated and measured doses in lung have been noted^69^, ^70^, ^71^ and can be worsened by user‐configurable parameters. It is worth noting that participation in clinical trials is no longer required to obtain such evaluation on a fee‐for‐service basis. Once an energy/algorithm is appropriately validated, facilities may rely on internal end‐to‐end tests for other clinically implemented energies and algorithms for the same TPS. If a formal third‐party mail‐in dosimetry evaluation is not possible, the results of the end‐to‐end tests should, at minimum, be peer‐reviewed by an independent QMP.

### 7.2 Recommendations

1. The range of optimization parameters (e.g., amount of modulation, minimum field size) and types of plans tested during commissioning should be clearly documented and representative of clinical practice. As clinical practices change, it may be necessary to conduct additional validation to accommodate new planning techniques.
2. Sufficient time should be devoted to fine tuning the MLC model parameters for the highly modulated plans.^61^ Procedures such as those developed by Saez et al.,^72^ Van Esch et al.,^73^ and Boeges et al.^74^ can be used to aid in the optimization of MLC model parameters.
3. A systematic bias has been observed in the TPS calculation of output for Test 7.2, which is most pronounced for lower energy beams (6 MV) and tertiary MLCs. For a 2 × 2 cm2 MLC‐defined field within a 10 × 10 cm^2^ jaw‐defined field, an average overestimation of output by about 3% has been reported.^29^, ^32^ A similar but smaller bias also exists for 3 × 3 cm^2^, although less pronounced.^29^, ^32^, ^36^ At this point, there are only inconsistent solutions available to the QMP; TPS manufacturers are encouraged to rectify this disagreement or provide commissioning tools to the physicist to improve agreement. In the meantime, a 2% tolerance for 3 × 3 cm^2^ or smaller fields in Test 7.2 may not be achievable. The QMP should aim to get optimal agreement for these output factors given the limitations and expectations referenced above.
4. The recommended evaluation criteria provided in Table 9 refer to true composite dose distributions that are recommended in TG‐218.^41^
5. The average difference between ion chamber and TPS doses across the low‐gradient target region of each plan should not exceed 2%, with less than 1.5% preferred.^65^ In higher gradient regions such as organs at risk (OAR), TG‐119 findings (agreement within 3% of prescription dose) are generally appropriate. The locally normalized dose difference should also be evaluated for areas and patterns of disagreement.
6. Planar or volumetric measurements (film or an electronic detector array with appropriate effective resolution) should be performed using a 10% threshold dose, absolute dose mode, and global normalization per TG‐218.^41^ Results should be initially evaluated with 2%/2 mm gamma analysis to emphasize areas of disagreement. Application of a local 2%/2 mm gamma criterion can result in the discovery of easily correctable problems with IMRT commissioning that may be hidden in higher passing rates.^69^ Final pretreatment QA results should demonstrate a gamma passing rate > 95% with 3%/2 mm criteria and a 10% dose threshold.^41^
7. There is even less guidance on the optimal criteria for end‐to‐end anthropomorphic test accuracy. This report recommends that the institution strive for a 5% agreement.
8. Additional verification testing may be required for treatment scenarios specific to a given delivery modality or linear accelerator. For example, if the MLC carriage does not move during delivery, it may be necessary to split fields, which can produce repetitive MLC leaf junction patterns that are sensitive to MLC modeling parameters. In addition, the treatment couch should be accounted for during IMRT/VMAT treatments that contain beams delivered through the couch.^75^, ^76^

**TABLE 9:** VMAT/IMRT evaluation methods and criteria

Measurement method	Regions	Evaluation criteria
Ion Chamber	Low‐gradient target region	2% of prescribed dose
	High‐gradient (OAR) region	3% of prescribed dose
Planar/Volumetric Array	All regions	No minimum pass rate,^a^ but investigate areas that fail at 2%/2 mm^b^
End‐to‐End	Low‐gradient target region	5% of prescribed dose

^a^ The QMP should follow TG‐218 ^41^ to develop tolerances for pretreatment QA that are appropriate at their clinic.

^b^ Application of a local 2%/2 mm gamma criterion can result in the discovery of easily correctable problems with IMRT commissioning.

## 8 ELECTRON BEAM VALIDATION

The AAPM TG‐25 Report^13^ and its supplement AAPM TG‐70 Report^14^ provide extensive detail on electron dosimetry. This current report is based on the AAPM TG‐70 Report recommendation that “…treatment planning for electron beams should be CT data based, employ 3‐D heterogeneity corrections and, at a minimum, use [Pencil Beam] PB‐based algorithms.” The following validation tests are recommended for routine electron therapy generated with image‐based electron planning systems employing 3D dose calculation algorithms (PB and MC). The limited accuracy and use of PB algorithms are well documented.^77^, ^78^, ^79^, ^80^ Monte Carlo algorithms are becoming a common practice in commercially available systems^81^, ^82^, ^83^, ^84^ and are recommended.

As with photons, electron commissioning data should be collected in air and water as specified by the vendor.^19^ Once the beam has been optimally modeled in the TPS, additional validation tests should be conducted to test the system's ability to calculate isodose distributions in nonstandard setups (e.g., patient specific cutouts, oblique incidence, extended SSD, and heterogeneous media). Much of the data for these validation tests can be obtained in water at the same time as the standard field data (used for modeling) is acquired. Other suitable phantom/detector combinations, such as array detectors, may also be used for validation measurements with consideration of the limitations of each device for electron dosimetry.^85^ The QMP should balance the advantages and disadvantages of different measurement devices when performing the following validation tests.

### 8.1 Validation tests

Four tests are summarized in Table 10 for validation of the TPS electron dose calculation algorithm. Test 8.1 is analogous to photon test 5.1 for electrons, whereby a beam equivalent to the beam calibration geometry should be planned in the TPS to ensure that the dose per MU matches the measured value at the calibration depth.

**TABLE 10:** Basic TPS validation tests for electron beams and minimum tolerance values

#	Dose comparison objective	Examples	Tolerances
8.1	Verify that TPS reproduces the absolute dose under reference calibration conditions	Calculate the absolute dose per MU to the reference point using the same SSD and cone size as the calibration protocol	0.5%
8.2	Basic model verification with shaped fields	PDD and profile comparison of custom cutouts at standard and extended SSDs	3%/3 mm
8.3	Surface irregularities/obliquity	PDD and/or profile comparison of obliquely incident fields using reference cone and nominal clinical SSD	5%
8.4	Inhomogeneity test	Compare manual dose calculation or chamber measurement to TPS with reference cone and nominal clinical SSD	7%

Test 8.2 compares the calculated isodose distribution for a custom shape cutout to a measured distribution at a standard and extended SSD. The cutout field size must be large enough to provide lateral scatter equilibrium.^14^ This will test both the system's ability to calculate dose with a custom cutout and verify that the virtual/effective SSD calculation is being applied correctly. This test should be performed for all energies.

Test 8.3 compares measured to calculated isodose distributions for an obliquely incident beam. This will test the impact of central axis tilt on depth dose and penumbra. This test should be performed in a homogeneous medium at the nominal clinical SSD for all energies.

Test 8.4 tests the electron dose calculation algorithm in the presence of heterogeneities. A calculation setup like the photon heterogeneity test described in Section 6 can be used. At a minimum, this test should be performed for energy at a suitable depth. Dose distributions from the TPS should be qualitatively compared to expected values.

### 8.2 Recommendations

1. Plot PDD and output factors for all cones (with standard cutout sizes) for each energy to confirm the correct qualitative behavior as a function of field size and energy.
2. For normal incidence (Test 8.2), measured and calculated isodose distributions should be within 3% agreement in the high‐dose region/low‐dose gradient and 3 mm DTA for PDDs along the central axis (excluding the buildup region). Note that percentages are of the central ray normalization dose.
3. For oblique incidence (Test 8.3), measured and calculated isodose distributions should agree within 5% in the high‐dose, low‐dose gradient region.^86^
4. Heterogeneity corrected manual dose calculations (Test 8.4) for the institution's heterogeneous phantom should be compared with CT‐based calculations.^3^ This comparison is generally qualitative, but the dose disagreement should not exceed more than 7%.
5. Clinically used nonroutine electron setups (e.g., abutting electron/electron fields, electron/photon fields, and small fields that results in a loss of lateral electron equilibrium) will require additional dosimetric verification to understand the limits of the electron dose model under these specific conditions.^87^

## 9 ESTABLISHING ROUTINE QA

Once commissioning has been completed, the QMP should establish a routine QA program to ensure that (1) the TPS has not been unintentionally modified and (2) dose calculation is consistent following TPS upgrades. Unintentional modifications can be identified using file integrity checksums.^1^ File integrity checksums use a computation algorithm that can periodically be run on a set of files to verify that their contents have not been altered. For TPSs, checksums are run on all executable, library, and other configuration or database content that is used for dose calculation. TPS vendors should provide methods for performing checksums on their respective systems. Dose calculation consistency can be performed by recalculating a subset of the tests defined in Sections 5, 6.2, 7, 8 of this report. This report recommends these tests be conducted annually or following TPS system upgrades.

Routine TPS QA complements machine QA, which validates the integrity of the linac output, MLC position, and other delivery parameters. Measurements are not required for TPS QA. Rather, each sample plan should be recalculated and compared to the baseline obtained during commissioning. Additional TPS checks, such as DVH calculation, effective depth calculation, and CT number consistency, can be performed using the same datasets.

### 9.1 Recommendations

1. Reference plans should be selected at the time of commissioning and then recalculated for routine QA comparison.
2. For photons, representative plans for each configured beam should be chosen from Table 5 for static and wedge beams and Table 8 for IMRT/VMAT.
3. For electrons, sample plans should be calculated for each energy using a heterogeneous dataset with reasonable surface curvature. It is also recommended to include extended distance and bolus verification in the sample plans.
4. Optionally, an additional thorax dataset with contours and suggested static beam parameters is included with the downloadable sample datasets (https://www.aapm.org/pubs/MPPG/TPS/). The curvature and inhomogeneity conditions of this dataset are applicable for TPS dose algorithm testing of wedged fields, dynamic arc, and/or electron plans.
5. All routine QA recalculations should agree with the reference dose calculation to within 1%/1 mm. A partial or complete recommissioning (including validation) may be required if more significant deviations are observed.

## 10 SUMMARY

The guidelines and recommendations provided in this report should aid the QMP in beam data acquisition, modeling, validation, and establishment of baseline routine QA datasets for their TPS. The QMP can substitute, alter, or add to the recommended test suite as needed if the change is made in the spirit of fulfilling or exceeding the minimum practice guidelines described within each section and justification is appropriately documented. As with all MPPG, this current report summarizes only a minimum scope of work that is necessary in the clinical setting.

Through completion of these guidelines, the QMP should also have improved his/her understanding of the strengths and limitations of the TPS beam model and dose calculation algorithms. Many TPS vendors provide guidance on expected levels of accuracy under different scenarios. The QMP should understand why these limitations exist and use them as a guide when evaluating the accuracy of their beam model. Knowledge of these limitations can also help define under what clinical applications the TPS is appropriate, and if new applications will require additional fine‐tuning or adjustment to the beam model.

Through the entire commissioning process, it is imperative to maintain clear and thorough documentation of the tests performed, equipment used, results, and findings. This documentation must be compiled into a final commissioning report by the QMP and appended with future TPS modification or recommissioning documentation. Appendix A is an optional Dose Algorithm Commissioning Checklist that can assist the QMP in determining whether the major tasks outlined in this document have been accomplished. It can also serve as a guideline for documentation.

Finally, the importance of peer review should be reiterated. Peer review of the TPS model parameters, agreement to measured data, and validation procedures/results is highly recommended. This should include independent dose calculations of basic dosimetry parameters (determined by another physicist) compared with independent measurements (also made by the other physicist).

## APPENDIX A Dose Algorithm Commissioning Checklist

Purpose: This optional commissioning checklist can assist the QMP in determining whether the major MPPG tasks have been accomplished and appropriately documented in the commissioning report. This inventory should be used only after the report has been read in its entirety.

Definition: A beam is typically distinguished as a unique energy and/or accelerator head model configuration. Each physical wedge or universal wedge is a unique beam because of its unique energy fluence spectrum and should be separately validated.

**Table:**

**#**	**Objective**	**Tolerances**	**In Report**
1‐2	QMP understands algorithms has received proper training		
2	Develop schedule to acquire data, model and verify the dose algorithms		
3	Consult manufacturer's guidance for data acquisition		
3.A	Verify operation, calibration of measurement equipment		
3.B	Acquire appropriate CT calibration data		
3.C	Review detector and corrections needs for small field measurements		
3.D	Review raw data (compare with published data, check for error, confirm import into TPS)		
4	Model per manufacturer's instructions, compare to published data for same machine/TPS		
5.1	Verify that TPS reproduces the absolute dose under reference calibration conditions	0.5%	
5.2	Compare calculated dose in clinical planning module to calculated dose in physics module	Within statistical, grid size, and material definition uncertainty	
5.3	Perform spot checks comparing calculated dose in clinical planning module to commissioning data	2%	
5.4	Small static (non‐SRS) MLC‐shaped fields	2% in high dose region (one parameter change) 5% in high dose region (multiple parameters) 2 mm in penumbra 3% of max dose in low dose tail (up to 5 cm from field edge)	
5.5	MLC transmission, leaf overtravel, output factor effects		
5.6	Off axis modelling including primary collimator		
5.7	Field divergence and depth dose changes with SSD		
5.8	Oblique surface incidence		
6.1	Validate planning system reported electron (or mass) densities against known values		
6.2	Heterogeneity correction distal to lung tissue	3%	
7.1	Verify small field PDD	2% of prescribed dose with ion chamber in low‐gradient region 3% of prescribed dose with ion chamber in high‐gradient region Evaluate planar/volumetric results at 2% local/2mm, pre‐treatment QA should pass >95% using 3% global/2mm and 10% threshold 5% of prescribed dose with end‐to‐end tests in low‐gradient region	
7.2	Verify output for small MLC‐defined fields		
7.3	TG‐119 tests		
7.4	Clinical tests		
7.5	External review		
8.1	Verify the TPS reproduces the absolute dose under reference calibration conditions for electron energies	0.5%	
8.2	Basic electron model verification with shaped fields	3%/3 mm	
8.3	Electron surface irregularities/obliquity	5%	
8.4	Electron inhomogeneity test	7%	
9	Routine QA established, including baseline QA plan(s) identified for each configured beam		
